# Retinopathy of prematurity: risk factors for its development in two neonatal intensive care units in Paraná-Brazil

**DOI:** 10.5935/0004-2749.20220049

**Published:** 2025-08-21

**Authors:** Sylvia M. F. Mayer, Larissa K. G. Mazarollo, Cristina Okamoto, Luciane Moreira, Luisa M. Hopker

**Affiliations:** 1 Hospital Universitário Mackenzie de Curitiba, PR, Brazil; 2 Hospital de Olhos do Paraná, Curitiba, PR, Brazil; 3 Complexo Hospitalar do Trabalhador, Curitiba, PR, Brazil

**Keywords:** Retinopathy of prematurity, Infant, newborn, Infant, premature, Infant, premature, diseases, Blindness, Retinopatia da prematuridade, Recém-nascido, Recém-nascido prematuro, Doenças do prematuro, Cegueira

## Abstract

**Purpose:**

Evaluate the patients in two neonatal intensive care units in Paraná/Brazil and identify the risk factors for the development of retinopathy of prematurity.

**Methods:**

We performed a prospective cohort study on premature infants with gestational age ≤32 wk and/or with birth weight ≤1500 g who were admitted to the neonatal intensive care unit of *Hospital do Trabalhador* and *Hospital Infantil Waldemar Monastier*. These hospitals admit patients referred from other maternity hospitals in the state of Paraná. The study duration was 12 mon.

**Results:**

The incidence of retinopathy of prematurity was higher in the *Hospital Infantil Waldemar Monastier* than in the *Hospital do Trabalhador* for premature infants who needed to be transported from their birthplace to the intensive care unit (52.2% vs. 29.6%). The following risk factors were associated with the development of the disease: longer hospitalization, low gestational age at birth, longer oxygen use, vasoactive drugs use, no antenatal corticosteroids use, intracranial hemorrhage, and any glycemic disorder. Low birth weight was an independent risk factor for the development of retinopathy of prematurity.

**Conclusion:**

Early neonatal care and transportation of premature infants may influence the occurrence and prognosis of retinopathy of prematurity.

## Abstract (pt)

**Objetivos:**

Avaliar duas unidades de terapia intensiva neonatais do Paraná e identificar os fatores de risco que levam ao desenvolvimento da retinopatia da prematuridade nestas unidades neonatais.

**Metodos:**

Foi realizado um estudo de coorte, prospectivo, com avaliação dos bebês prematuros examinados no período de 12 meses com idade gestacional ≤32 semanas e/ou com peso de nascimento ≤1500 gramas, internados na unidade de cuidados intensivos neonatais do Hospital do Trabalhador e do Hospital infantil Waldemar Monastier, que recebe neonatos transportados das maternidades de todo o estado do Paraná.

**Resultados:**

A incidência de retinopatia da prematuridade foi maior no Hospital Infantil Waldemar Mo nastier, entre os prematuros que necessitaram de transporte do local de nascimento para a unidade de cuidados intensivos (52,2% vs 29,6%), Os fatores de risco associados ao desenvolvimento da doença foram; Maior número de dias de internamento, baixa idade gestacional ao nascimento, maior tempo de uso de oxigênio, uso de drogas vasoativas, ausência de uso de corticoide pré-natal, presença de hemorragia intracraniana e qualquer tipo de alteração da glicemia.

**Conclusão:**

Os cuidados neonatais precoces e o transporte do recém-nascido prétermo podem influenciar a ocorrência e o prognostico da retinopatia da prematuridade.

## INTRODUCTION

Retinopathy of prematurity (ROP) was first described by Terry in 1942^(1)^. In 1951, Health P. called it ROP^(2)^. ROP is a vasoproliferative disease secondary to the inadequate vascularization of the immature retina of premature newborns (NPMs)^(3,4)^, which can trigger serious visual sequelae^(5,6)^.

The main factors that influence its development are low gestational age, low birth weight, blood transfusion, prolonged oxygen therapy^(7)^, intracranial hemorrhage^(8)^, prolonged stay in the neonatal intensive care unit (NICU), and the presence of infection^(8,9)^.

Care in the first hour of life exerts a strong influence on several of these factors and consequently in ROP^(3,8,10,11)^, especially in NPMs that are transported to other units^(12,13)^. Among several factors, this care contributes to the differences in the prevalence of this disease between developed and undeveloped countries^(3)^.

Screening for NPMs and timely treatment prevents potential complications in up to 50% of the patients^(3)^.

In Brazil, the significance of ROP has increased owing to improvements in the survival of NPMs; in 2002, the first screening protocol for ROP was defined after a symposium with several groups committed to the treatment and prevention of the disease^(14)^.

## METHODS

This was a cohort, prospective, descriptive, quantitative study. Premature babies who were born at ≤32 wk of gestational age (GA) and/or with birth weight ≤1500 g were examined from the fourth week of life to the 46 weeks corrected age; we examined those infants who were admitted to the NICU of the *Hospital do Trabalhador* (HT) and *Hospital Infantil Waldemar Monastier* (HIWM) over the 12-month period from May 2013 to May 2014. The following exclusion criteria were applied: NBs who died before the first fundus examination or before completing 42 wk of corrected GA and NBs who were discharged and were not brought for the ophthalmological follow-up.

Ophthalmological examinations were performed as per the Brazilian guidelines for the examination and treat ment of ROP^(5)^, and staging was performed as per the International Classification of ROP (ICROP)^(15)^.

The following variables were analyzed: sex, 5-minute APGAR score, antenatal corticosteroids use, birth weight, GA at birth, oxygen-therapy duration, vasoactive drug use, intracranial hemorrhage, neonatal infection, glycemic disorder, hospitalization duration, and need for blood transfusion.

The mean, median, minimum value, maximum value, and standard deviation were considered to describe the quantitative variables. To summarize the qualitative variables, frequencies, and percentages. To compare two classifications of a variable in relation to a quantitative variable, Student’s t tests were used. Further, for independent samples, the non-parametric Mann-Whitney test was used. To assess the association of qualitative variables, the Chi-square test, and the exact Fischer test were performed. For joint assessment of variables with the presence of ROP, a logistic regression model was applied, and for assessing the normality of quantitative variables, the Jarque-Bera test was performed. P-values <0.05 indicated statistical significance. The weight gain ratio formula was as follows: (weight weeks - birth weight)/birth weight.

## RESULTS

During the study period, 464 patients were evaluated, including 46 patients from the HIWM and 54 from HT who met the inclusion criteria. Of these, 47 (47%) were women and 53 (53%) were men (p=0.173). The results were analyzed by comparing the two NICUs (HIWM and HT).

There was a significant difference in the development of ROP between the two hospitals (p=0.022). ROP (any stage) was present in 24 (52.2%) patients of the HIWM and 16 (29.6%) of the HT; ROP grade 3 was present in 9 (37.5%) infants at the HIWM and 3 (18.8%) at the HT (Table 1).

**Table 1:** Comparison on the degree of ROP between the hospitals

Degree of ROP^*^	H1WM N (%)	HT N (%)
1	6 (25.0)	6 (37.5)
2	9 (37.5)	7 (43.8)
3	9(37.5)	3(18.8)
Total	24(100.0)	16(100.0)

p-value= 0.423.

* Restricted to patients who developed ROP.

HT= *Hospital do Trabalhador*; HIWM= *Hospital Infantil Waldemar Monastier*.

There was no difference in the degree of the disease or the development of plus disease between both the NICUs (p=0.423 and p=0.136, respectively) (Tables 1 and 2).

**Table 2:** Comparison of hospitals with respect to the presence of plus disease associated with ROP

Plus disease^*^	HIWM N (%)	HT N (%)
Yes	8 (33.3)	2 (12.5)
No	16 (66.7)	14 (87.5)
Total	24 (100.0)	16 (100.0)

p-value= 0.136.

* Restricted to patients who developed ROP.

HT= *Hospital do Trabalhador*; HIWM= *Hospital Infantil Waldemar Monastier*.

Two patients (12.5%) at the HT and 8 (33.3%) at the HIWM had severe ROP and required treatment (laser and antiangiogenic) (Table 3); it was not possible to perform the statistical analysis owing to the small samples sizes.

**Table 3:** Outcomes in patients with ROP

Outcomes in patients with ROP	HIWM N (%)	HT N (%)
Spontaneous regression	14(58.3)	13 (81.3)
Laser	7(29.1)	2 (12.5)
Bevacizumab	1 (4.2)	0 (0.0)
Follow-up lost	2 (8.4)	1 (6.2)
Total	24(100.0)	16 (100.0)

HT= *Hospital do Trabalhador*; HIWM= *Hospital Infantil Waldemar Monastier*.

There was no significant difference in the birth weight, GA, and oxygen-therapy duration between the groups (p=0.860, p=0.983, and p=0.168, respectively) (Table 4). Not even when relating ROP to sex, APGAR of the fifth minute, presence of acquired infection and need for blood transfusion (p=0.234, p=0.749, p=0.231, and p=0.881, respectively) (Tables 4 and 5).

**Table 4:** Comparison between development of ROP and the qualitative variables at the maternities

Variables	Hospital	N	Average	Median	DP	p-value
Hospitalization duration (d)	HIWM HT	46 53	79.5 55.5	72.0 50.0	40.9 30.6	0.002^**^
Birth weight (g)	HIWM	46	1226.8	1092.5	383.9	0.860^*^
	HT	54	1239.6	1270.0	338.3	
Gestational age (wk)	HIWM	46	29.4	30.0	2.3	0.983^*^
	HT	54	29.4	30.0	2.2	
5-minute APGAR score	HIWM	44	8.3	9.0	1,5	0.749^**^
	HT	52	8.2	8.5	1,6	
O2 time (DAYS)	HIWM	45	48.9	37.0	48.7	0.168^**^
	HT	49	31.0	23.0	29.2	

* Student test for independent quantities; p<0.05.

** Mann-Whitney non-parametric test; p<0.05.

HT= *Hospital do Trabalhador*; HIWM= *Hospital Infantil Waldemar Monastier*.

**Table 5:** Association between comorbidities and ROP

Morbidities		HIWM N (%)	HT N (%)
Intracranial hemorrhage	Yes	35 (76.1)	18 (34.0)
	No	11 (23.9)	35 (66.0)
	Total	46 (100.0)	53 (100.0)
p-value	<0.01		
Received vasoactive drugs	Yes	12 (26.1)	25 (47.2)
	No	34 (73.9)	28 (52.8)
	Total	46 (100.0)	53 (100.0)
p-value	<0.031		
Presence of glycemic disorders	Yes No	29 (63.0) 17 (37.0)	16 (30.2) 37 (69.8)
	Total	46 (100.0)	53 (100.0)
p-value	0.001		
Glycemic disorders	Hyperglycemia	5 (17.24)	11 (68.75)
	Hyperglycemia + hypoglycemia	5 (17.24)	1 (6.25)
	Hypoglycemia	19 (65.52)	4 (25.0)
	Total	29 (100.0)	16 (100.0)
p-value	0.003		
Antenatal use of corticosteroids	Yes No	18 (39.1) 28 (60.8)	34 (63.0) 20 (37.0)
	Total	46 (100.0)	54 (100.0)
p-value	0.017		
Acquired infection	Yes	1 (2.18)	4 (7.4)
	No	45 (97.8)	50 (92.6)
	Total	46 (100.0)	54 (100.0)
p-value	0.231		
Blood transfusion	Yes	35 (76.1)	42 (77.7)
	No	11 (23.91)	12 (22.22)
	Total	46 (100.0)	54 (100.0)
p-value	0.881		

HT= *Hospital do Trabalhador*; HIWM= *Hospital Infantil Waldemar Monastier*.

The groups were significantly different in terms of hospitalization duration and the ROP development (p=0.002); the length of hospitalization was 79.5 d at the HIWM and 55.5 d at the HT (Table 4). There was also a difference in the corrected GA at the most recent eye examination; it was 45.7 wk in the HIWM and 42.1 wk in the HT (p=0.004).

Moreover, we found a correlation of ROP development with the absence of antenatal corticosteroids use, intracranial hemorrhage, vasoactive drugs use, and glycemic disorder (p=0.017, p < 0.001, p=0.031, and p=0.001, respectively) (Table 5).

Among the glycemic disorders there was a significant difference in the groups (p=0.003). At the HIWM, an equal percentage of patients developed isolated hyperglycemia and hyperglycemia associated with hypoglycemia (17.24%), and at HT, 68.75% presented isolated hyperglycemia.

## DISCUSSION

Blindness due to ROP has been reported since >70 y; in the 1990s, it was linked to an increase in the number of visually impaired people with higher requirement of NICU beds; better early neonatal care resulted in an increase in the survival rates^(16-18)^.

Today, being an important cause of global blindness, it is mainly concentrated in countries that have not standardized early detection and treatment of ROP^(17,19)^. The NICUs analyzed differ in the origin of their patients. At HT come from the hospital´s own maternity and the HIWM receives patients born in other hospitals

around the state.

There was a significant difference between the two groups with respect to the development of ROP (p=0.022). Regarding the degree of ROP development and the presence of plus disease, there was no significant difference between the groups (p=0.423 and p=0.136, respectively). We found a discrepancy in the development of the pathology between the groups considering that both had the same infrastructure, received equally complex NPMs, and had a homogeneous population in relation to sex (p=0.234), birth weight (p=0.860), GA at birth (p=0.983), and 5-minute APGAR score (p=0.749); thus, the infants were expected to undergo similar development.

This difference in ROP development in the NICUs is attributable to the fact that HIWM patients come from different hospitals, receive immediate non-standardized postnatal care, and are transported to the referred NICU as per different protocols. However, in the HT, they follow the same postnatal care for all patients.

Management during the first hour of life is an important factor that influences the development of comorbidities in NPMs^(10,11,20)^. In 2017, Sharma D. reduced neonatal morbidities, including ROP, using a standard protocol for the first hour of life^(20)^. At the HIWM, where there was a higher incidence of ROP and treated ROP (52.2%, 33.3%, respectively) all of their NPMs were from different maternity hospitals and without the data corresponding to management in that first hour of life. In contrast, experienced pediatricians with a pre-established protocol treated the infants at the HT.

In a similar manner, the infants at the HIWM had a longer hospital stay (p=0.002) and more comorbidities, including ROP, necessitating prolonged ophthalmological follow-up and other care, the average of the last HIWM eye examination was 45.7 wk and at the HT was 42.1 wk.

Kuo et al.^(12)^ compared the infants at the NICUs from the same hospital and those transported from other centers; they observed higher rates of ROP and hospital stay in the transferred group, reinforcing the importance of management in the first hours of life. In that study, no hospital from which the patients performed oximetry to prevent ROP. They concluded that strict management of oxygen in the first hours of life before the arrival of transport could improve the ROP rates in these infants^(12)^. Chung et al.^(13)^ also analyzed the NPMs of the hospital and the NPMs transported to another center; however, they were transported within a short distance, and the transfer was performed by a trained team, complying with a pre-established protocol. The analyzed variables and results were similar to those in our study, showing a higher incidence of ROP and hospitalization in the transported group^(13)^. Multivariate analyses showed no differences in several comorbidities, including ROP^(13)^, potentially owing to the condition in which transport was performed.

Other factors linked to the development of ROP are oxygen-therapy use, blood transfusion, and infection, which showed no significant difference (p=0.168; p=0.881 and p=0.221), indicating that they follow similar behaviors in these aspects, in addition to the fact that certain infections are almost inevitable in NPMs^(21-23)^.

Referent to the prenatal use of corticosteroids, we have significant results (p=0.017), relating it as a protective factor for the development of ROP. In 2018, Yim et al.^(24)^ compared studies where prenatal steroids, such as console and cols, were used with a 65% and 93% reduction in the risk of development and progress of the ROP and Higgins and Cols. with 82% less chance of developing ROP stage 2 or higher and Yu-Shu Liu et al. with decreased incidence of severe ROP from 81.9% to 60%. Yim et al.^(24)^, concluding that the use of prenatal steroids reduces the risk of ROP development and it progress^(24)^.

In our study, the prevalence of prenatal corticosteroids use in HT patients with ROP was 63% and that in HIWM patients was 39.1%. The prevalence of corticosteroids use was 40.4%, while that of non-use was 39.6%; there was no significant difference in the two groups (p=0.935). We explain this discrepancy in the global rates because more patients in the HIWM group had ROP with less drug use; the HT group showed contradictory results, so when added, they do not have significant differences, however, to evaluate each service, HT with a greater use of corticosteroids and lower ROP rate, we observed and agreed with the importance of the drug.

The intracranial hemorrhage was significantly different (p<0.001), the HIWM had almost twice the pathology. It is believed that this fact also due to the fallacies in previous care and during transportation.

The use of vasoactive drugs was significantly higher (p=0.031) in the HT group, with 47.2% of those in the HT group using them because in this group, we know exactly what was being used for each infant. At the HIWM, different protocols were followed for the patients associated with possible data omission.

Glycemic disorders were significantly different between the groups (p=0.001), at HIWM (63%) presenting any disorder, while at the HT (30.2%), associating ROP with glycemic disorders but not showing a preference for some type of variation. This is because each service has different glycemic correction schemes and because these changes easily fluctuate in the NPMs.

The same finding was reported by Mohamed et al. who showed an association between the duration of hyperglycemia and the development of ROP^(25)^. Lee et al. reported that hyperglycemia is common in NPMs, associated with an increased incidence of mortality and morbidities, such as ROP^(26)^. In a similar manner, Jagła M et al. showed that big glycemic variations are associated with severe morbidities, such as ROP and necessitate treatment^(27)^. All these reports are in agreement with our report with respect to the association of glycemic disorders with ROP development.

We compared two neonatal NICUs; both had similar infrastructure and human resources, with the main difference being the origin of the patients. Whom were transported from other maternities to the HIWM and at the HT the patients were from the own maternity hospital. The differences in ROP incidences are justified because HIWM patients received diversified management in the first hours of life and at the time of transport until they reached the reference hospital. At HT, all the patients came from their own maternity, following the same care protocol, with a better outcome.

They also differed in the hospitalization duration and corrected GA at the last eye examination, being higher for the transported group, owing to the presence of more comorbidities and ROP, necessitating prolonged hospitalization. In a similar manner, there was a significant difference with respect to glycemic disorders and the presence of intracranial hemorrhage, with a higher percentage in this group too.

Other differences were the greater use of vasoactive drugs and prenatal corticosteroids in mothers at the HT (with lower rates of ROP) owing to their standardized conditions in the first hour of life and their well-established prenatal and maternal care with quick access to the hospital when complications developed.

The importance of this study is that it reinforces previous results^(3,10-13,28)^ with respect to the risk factors for ROP, emphasizing the importance of management during the first hour of life and the transportation of these patients.
